# Low serum levels of High-Density Lipoprotein cholesterol (HDL-c) as an indicator for the development of severe postpartum depressive symptoms

**DOI:** 10.1371/journal.pone.0192811

**Published:** 2018-02-14

**Authors:** Raji Ramachandran Pillai, Anand Babu Wilson, Nancy R. Premkumar, Shivanand Kattimani, Haritha Sagili, Soundravally Rajendiran

**Affiliations:** 1 Ph.D Scholar, Department of Biochemistry, Jawaharlal Institute of Post-graduate Medical Education and Research (JIPMER), Puducherry, India; 2 Junior Research Fellow, Department of Biochemistry, Jawaharlal Institute of Post-graduate Medical Education and Research (JIPMER), Puducherry, India; 3 Medical Social Worker, Medico Socio Services, Jawaharlal Institute of Post-graduate Medical Education and Research (JIPMER), Puducherry, India; 4 Additional Professor, Department of Psychiatry, Jawaharlal Institute of Post-graduate Medical Education and Research (JIPMER), Puducherry, India; 5 Associate Professor, Department of Obstetrics & Gynecology, Jawaharlal Institute of Post-graduate Medical Education and Research (JIPMER), Puducherry, India; 6 Additional Professor, Department of Biochemistry, Jawaharlal Institute of Post-graduate Medical Education and Research (JIPMER), Puducherry, India; Universitat de Lleida-IRBLLEIDA, SPAIN

## Abstract

Postpartum depression (PPD) is a psychiatric complication of childbirth affecting 10–20% of new mothers and has negative impact on both mother and infant. Serum lipid levels have been related to depressive disorders, but very limited literatures are available regarding the lipid levels in women with postpartum depression. The present study is aimed to examine the association of serum lipids with the development of postpartum depressive symptoms. This is a cross sectional study conducted at a tertiary care hospital in South India. Women who came for postpartum check-up at 6^th^ week post-delivery were screened for PPD (September 2014-October 2015). Women with depressive symptoms were assessed using EPDS (Edinburgh Postnatal Depression Scale). The study involved 186 cases and 250 controls matched for age and BMI. Serum levels of lipid parameters were estimated through spectrophotometry and the atherogenic indices were calculated in all the subjects. Low serum levels of Total Cholesterol (TC) and High Density Lipoprotein cholesterol (HDL-c) were significantly low in PPD women with severe depressive symptoms. The study recorded a significant negative correlation between HDL-c and the EPDS score in PPD women (r = -0.140, p = 0.05). Interestingly, the study also observed a significant negative correlation between Body Mass Index (BMI) and EPDS scores in case group (r = -0.146, p = 0.047), whereas a positive correlation between the same in controls (r = 0.187, p = 0.004). Our study demonstrated that low levels of serum HDL-c is correlated with the development of severe depressive symptoms in postpartum women. Study highlights the role of lipids in the development of postpartum depressive symptoms.

## Introduction

In women, pregnancy and postpartum period are regarded as the crucial periods of time for the occurrence of psychiatric disorders. Postpartum depression (PPD) is a psychological complication following child birth. PPD differs from ‘postpartum blues’ which is a brief period of mood disturbance that begins three to five days after childbirth and may last up to two weeks [1] and ‘postpartum psychosis’ which is a rare psychiatric episode that develops within the first two weeks after delivery and requires hospitalization [2]. PPD can persist for a short period and remit abruptly and about 50% of women with postpartum depressive symptoms continue to have major depression throughout beyond the first year post-delivery [3].Varying rate of occurrence of postpartum depressive symptoms have been reported in and across countries, which ranges from 10–42%, with higher rates in developing countries [4]. Despite its serious outcomes, PPD often remains unrecognized. If untreated and left undiagnosed, it leads to detrimental outcomes: affects the mother- child bonding, increases risk of infanticide and maternal suicide and adversely affects the child’s cognitive and behavioural development [5].

Alterations in maternal lipid metabolism occur during pregnancy and are associated with the gestational age. The maternal serum lipid levels increases during pregnancy and in the postpartum period [6]. An increase in serum levels of Triglycerides (TG) and Total Cholesterol (TC) has been observed in pregnant women with the progression of gestational age [7]. Previous studies have shown higher levels of Total Cholesterol, Low-Density Lipoprotein cholesterol (LDL-c) and High-Density Lipoprotein cholesterol (HDL-c) all of which remain elevated above pre-pregnancy during postpartum period [8]. These increased lipid levels during pregnancy serves as an energy reserve to fulfil both maternal and fetal metabolic demands and towards late gestational period, lipid levels seems to play important role in milk formation before parturition [9]. During late pregnancy, there is a significant drop of fat deposits which play a vital role in fetal development and growth.

Obesity has been found to be associated with depressive disorders. Also, maternal obesity in pregnancy is linked with negative outcomes such as gestational diabetes, preeclampsia in mothers as well as stillbirth and congenital anomalies in the fetus and also poses future risk for heart disease, hypertension and increased risk of obesity and heart disease in their offspring [10]. A study by Robert etal reported higher risk of depression in obese people [11] while others reported that there was no relation between obesity and depression [12].The psychological distress due to obesity also leads to depression. Additionally, previous studies have reported that both obesity and depression are influenced by socio-economic status as well as family history of depression [13]. Studies report that Asian Indians have higher body fat, waist to hip ratio, intra-abdominal fat compared to Caucasians. The difference may be attributed because of lifestyle and other epigenetic factors [14, 15].

The relationship of serum lipid levels with various psychiatric disorders including depression has been reported in previous studies [16, 17]. Pregnant women with depressive symptoms are found to be overweight and obese [18]. Serum cholesterol is found in increased amounts in the circulation of normal pregnant women. Additionally, High-Density Lipoprotein cholesterol (HDL-c) and Low-Density Lipoprotein cholesterol (LDL-c) have also been found to be associated with the development of depression [19, 20]. In support of this, a study by Wysokinski et al suggested that low serum levels of circulating lipids are identified as risk factors for depression even in patients suffering with other psychiatric illnesses like schizophrenia, unipolar and bipolar disorder [21]. Furthermore, elevated total cholesterol has been identified as a potent risk factor for cardiovascular disease (CVD) and early death [22]. Depression is identified as an independent risk factor for future CVD, which is the common cause of female morbidity and mortality [23]. Moreover, there is a lacuna about the role of lipid levels and atherogenic indices in postpartum mood disorders. In view of the above, we aimed to investigate the association of lipid profiles in postpartum women with and without depressive symptoms at six week post—delivery.

Several studies have implicated the relationship of serum lipid levels with depressive disorders. However, to the best of our knowledge, there is only one study available on postpartum lipid levels.There are no studies on the association of serum lipid levels as well as calculated atherogenic lipid indices with postpartum depression at six week post-delivery. Our study aimed to evaluate these at 6 weeks post-delivery in women with postpartum depressive symptoms.

## Materials and methods

This is a cross sectional study carried out in the Department of Biochemistry, Jawaharlal Institute of Post-graduate Medical Education and Research (JIPMER) hospital, Puducherry, South India during the period from September 2014 to October2015. The study was approved by the Institute Ethics Committee (Human Studies), JIPMER (Ref no. JIP/IEC/2014/5/319).

### Study population and outcome measures

The study subjects were women who came to the hospital for postpartum check-up at 6^th^ week post-delivery. A total of 696 participants were screened consecutively during the study period. Selection was based on as per the following criteria: no previous life time history of any endocrine dysfunction, neurological disorder, depressive or any psychiatric illness or use of antidepressant drugs. Existing mental classificatory systems require that to call depression as postpartum onset it has to have its onset within first 4 to 6 weeks post–delivery. Assessment at 6 weeks was chosen, as we wanted to choose those who developed depressive symptoms after delivery but before the 6 weeks post-delivery. By this, we excluded those with transient mood changes or those with postpartum blues, and those with prior history of depressive illness, or their residual symptoms, or whose depressive symptoms being modified by antidepressant use.

**Fig 1:**
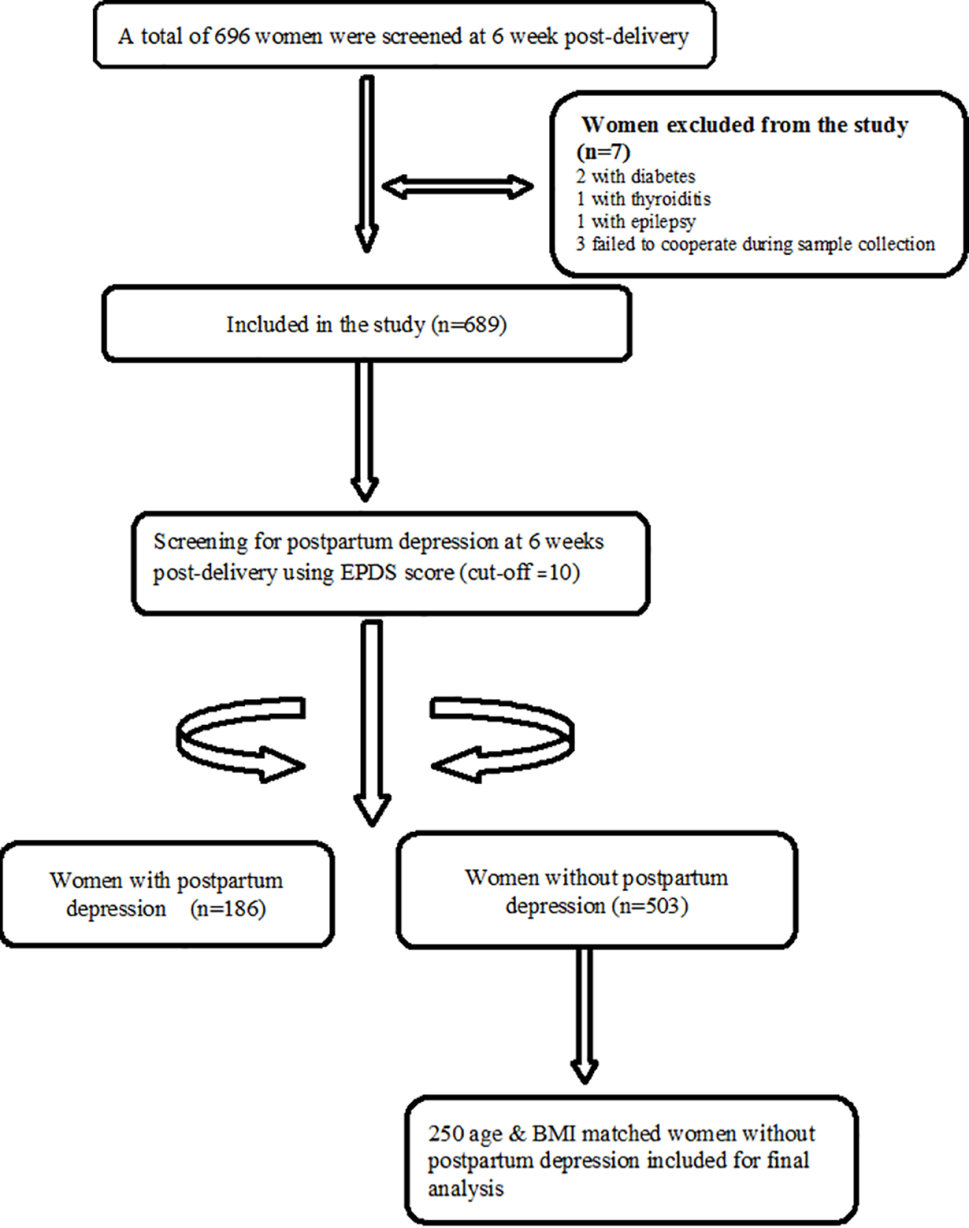
Flow chart of the study population.

After complete description of the study to the subjects, written informed consent was obtained from the participants before enrolment in the study. All the women were interviewed at the time of recruitment. A semi- structured interview was conducted with detailed questionnaire. The structured questionnaire included questions on socio-demographic, psychiatric history (previous history of depression or psychiatric illness or anytime on antidepressant drugs), gynaecological, obstetric history details, lifestyles and dietary details. Tamil version of Edinburgh Postnatal Depression Scale (EPDS) was used to assess depression in postpartum women at the time of visit. Clinical data and neonatal outcome details were gathered from medical records. EPDS is a widely used screening questionnaire for the detection of postnatal depression [24]. It is a 10 item questionnaire related to depressive symptoms in the past one week and each item is rated on a four point likert scale. EPDS score ranges from (0–30), cut off score of 10 and above has sensitivity of 76.5% and specificity of 92.9% to detect PPD as made by ICD-10 classification [25]. Study subjects were allocated into two groups with PPD (cases) and without PPD (controls) on the basis of EPDS score. From 689 subjects included in the study, 186 women had scores ≥ 10 were enrolled as cases. Controls were 250 postpartum women without PPD and were matched for age and BMI. The PPD women were categorized based on their EPDS median score of 12 as high scores (≥12) are generally accepted as strong indicator of major or minor depressive symptoms. The study used a cut-off of 10 to define the PPD cases among South Indian population as per Benjamin et al, which is considered a best fit for local population [25]. However for better understanding, the PPD women in the study were categorized based on their EPDS median score of 12 as high scores (≥12).We performed the subgroup analysis of anthropometric measures and serum levels of lipid parameters as in order to examine significant difference between serum lipid parameters in postpartum depressive and non- depressive women based on this median EPDS score. The suicidal risk in postpartum depressive women was examined by performing the subgroup analysis of these women based on suicidal ideation or thoughts of self-harm. Based on the previous available reports of suicidality in the postnatal period, the suicidal ideation was assessed by examining the item no. 10 of the EPDS which is denoted as “The thought of harming myself has occurred to me”. Possible responses included ‘never’ = 0, ‘hardly ever’ = 1, ‘sometimes’ = 2, or ‘quite often’ = 3 [26, 27]. Women with high scores (EPDS ≥ 20) or any thoughts of self -harm (EPDS item 10 ≥ 1) were considered to have suicidal ideation. Women who had high scores (EPDS ≥ 20) or any thoughts of self-harm (EPDS score on item no.10 ≥ 1) were allocated into the group of PPD women with suicidal ideation (N = 45). Flow chart showing the overall recruitment of study subjects, case–control selection is depicted in Fig 1.

### Blood sampling and analysis of lipid profile

Peripheral venous blood samples (5ml) were collected from all the study subjects at the time of initial assessment. Samples were centrifuged to separate the serum at 5000 rpm for 10 minutes at room temperature and fresh serum was used immediately for the analysis of lipid parameters. The estimation of serum Total Cholesterol (TC), Triglycerides (TG), High Density Lipoprotein cholesterol (HDL-c) was carried out in an automated clinical auto analyser (Beckman coulter AU 680) within 24 hrs. from the time of sampling. Further Low Density Lipoprotein cholesterol (LDL-c),Very Low Density Lipoprotein cholesterol (VLDL-c) and non-HDL-c were calculated by using Friedewald’s formula [28].The atherogenicratios like, Castelli’s risk index (CRI-I = TC/HDL-c and CRI-II = LDL-c/HDL-c), atherogenic coefficient (AC) = (TC–HDL-c)/HDL–c, atherogenic index of plasma (AIP) = log (TG/HDL-c) were calculated for individual subjects [29]. Maternal height and bodyweight at six week post-delivery were obtained from medical records of the subjects at the time of postpartum clinic visit. From these variables, Body Mass Index (weight (kilograms)/height (meters squared) was calculated and recorded.

### Statistical analysis

All categorical and qualitative variables were presented as frequencies and percentages. Normality of the continuous data was tested by Kolmogorov–Smirnov test and accordingly appropriate parametric or non-parametric tests were used. Non- Gaussian data was presented as median with inter-quartile range. Mann-Whitney U test was used for the comparison of serum lipid levels between cases and controls as well as the comparison of lipid profile based on EPDS median score. Chi-square test was used to compare the socio-demographic data between the groups. For correlation studies, Spearman correlation analysis was used to assess the linear relationship between serum lipid parameters with EPDS score. All statistical analysis was carried out at 95% confidence interval and p value <0.05 considered significant. All statistical analysis was carried out using SPSS version 19 (Armonk, NY: IBM Corp).

## Results

### Anthropometric measures and lipid profile in postpartum women at 6 week post-delivery based on EPDS cut- off score

The anthropometric and lipid profile of postpartum women with and without depressive symptoms based on EPDS cut–off score 10 are expressed as median (Interquartile Range) and represented in Table 1. There was no significant difference in serum levels of lipid parameters between the two groups.

**Table 1:** Anthropometric measures and serum lipid levels expressed as median (Interquartile Range) at 6^th^ week postpartum in women with and without depressive symptoms based on EPDS cut–off score 10.

Parameters	EPDS (10)		P value
	EPDS <10 (N = 250)	EPDS ≥10 (N = 186)	
Age (yrs)	24 (22–28)	25 (23–29)	0.172
BMI (kg/m^2^)	24.75 (22.40–28.83)	24.78 (22.19–27.84)	0.621
Total Cholesterol (mg/dl)	198 (174–222)	202 (179.75–225.25)	0.363
Triglyceride (mg/dl)	117 (79.50-158-50)	109 (83–155)	0.579
HDL-c (mg/dl)	49 (43–56)	50 (42.75–56.25)	0.976
LDL-c (mg/dl)	121 (103–141)	124 (107–143.25)	0.125
VLDL-c (mg/dl)	23 (16–32)	22 (17–31.50)	0.657
Non HDL-c (mg/dl)	148 (127–170)	150 (132–169.25)	0.441
Atherogenic Coefficient	3.02(2.57–3.48)	3.02 (2.66–3.53)	0.541
(TG/HDL) ratio	2.33 (1.61–3.45)	2.17(1.62–3.29)	0.739
AIP	0.37 (0.22–0.54)	0.35 (0.24–0.53)	0.821
Castelli Index 1 (CRI-I)	4.02 (3.57–4.46)	4.02 (3.66–4.53)	0.547
Castelli Index 2 (CRI-II)	2.45 (2.11–2.91)	2.51 (2.18–2.86)	0.246

HDL- c: High density lipoprotein cholesterol, LDL- c: Low density lipoprotein cholesterol VLDL-c: Very low density lipoprotein cholesterol, AIP: Atherogenic Index of Plasma

EPDS: Edinburgh Postnatal Depression Scale, PPD: Postpartum depression

Statistical analysis using Mann Whitney U test

### Serum lipid levels in PPD women at six week postpartum based on median EPDS score

The subgroup analysis of anthropometric measures and serum levels of lipid parameters in PPD women categorized based on their EPDS median score are shown in Table 2. No statistical significant difference was observed in median levels of anthropometric variables and serum lipid levels between the two groups except for the Castelli’s risk indices. The serum HDL-c levels were observed to be lower in PPD women with EPDS score greater than 12 compared to PPD women with EPDS score less than or equal to 12 without any statistical significance. Although the median levels of serum lipid levels were lower in postpartum women with EPDS score of more than 12 except for CRI-I and CRI-II levels, their difference didn’t show any statistical significance using Mann Whitney U-test. Meanwhile Castelli’s risk indexes I [4.09 (3.78–4.64) vs. 3.96 (3.53–4.36), p = 0.036)] and II [2.60 (2.29–2.77) vs. 2.42 (2.08–2.79) p = 0.015)] were found to be significantly elevated in PPD women with EPDS score greater than 12 compared to those with less than 12 EPDS score.The Castelli’s risk indices were studied as markers of lipid atherogenic risk and are used in clinical scenario for assessing the risk of CVD.

**Table 2:** Serum lipid levels expressed as median (Interquartile Range) in PPD women group categorized based on median EPDS score.

Parameters	Median of EPDS score (12)		P value
	≤12 (N = 91)	>12 (N = 95)	
Age (yrs)	25 (22–29)	26 (23–29)	0.415
BMI (kg/m^2^)	25.10 (22.40–29.00)	24.20 (22.10–27.40)	0.433
Total Cholesterol (mg/dl)	202 (184–223)	198 (174–226)	0.565
Triglyceride (mg/dl)	109 (84–155)	107 (81.50–153)	0.793
HDL-c (mg/dl)	51 (45–57)	49 (41–55)	0.050
LDL-c (mg/dl)	125 (107–142)	122 (107–150)	0.666
VLDL-c (mg/dl)	22.50 (17–32.25)	22 (16–29)	0.430
Non HDL (mg/dl)	152 (132–168)	150 (131–175)	0.820
AC	2.96 (2.53–3.36)	3.08 (2.77–3.59)	0.087
(TG/HDL) ratio	2.18 (1.61–3.21)	2.15 (1.62–3.43)	0.788
AIP	0.36 (0.24–0.52)	0.35 (0.23–0.55)	0.871
Castelli Index 1 (CRI-I)	3.96 (3.53–4.36)	4.09 (3.78–4.64)	0.036*
Castelli Index 2 (CRI-II) (CRI-II)	2.42 (2.08–2.79)	2.60 (2.29–2.77)	0.015*

HDL- c: High density lipoprotein cholesterol, LDL- c: Low density lipoprotein cholesterol VLDL-c: Very low density lipoprotein cholesterol, AIP: Atherogenic Index of Plasma

AC: Atherogenic Coefficient

EPDS: Edinburgh Postnatal Depression Scale, PPD: Postpartum depression

<0.05 is significant

*statistical significance using Mann Whitney U test

### Serum lipid levels in PPD women based on suicidal ideation

The serum lipid levels of depressive women with suicidal ideation and without suicidal ideation are represented in Table 3 as Median (Interquartile Range). The suicidal ideation was assessed as mentioned in the methodology section. The median serum levels of TC and HDL-c were significantly lower in women with suicidal ideation when compared to women without suicidal ideation. But, no significant difference was observed in serum levels of other lipid parameters.

**Table 3:** Serum lipid levels expressed as median (Interquartile Range) in PPD women with and without suicidal ideation.

Parameters	PPD women without Suicidal Ideation (N = 141)	PPD women with Suicidal Ideation (N = 45)	P value
Total Cholesterol (mg/dl)	202 (185–225.50)	181(166.50–222)	0.032*
Triglyceride (mg/dl)	111 (86.50–155)	101(75.50–172.50)	0.469
HDL-c (mg/dl)	51 (45–57)	47 (40–51)	0.005*
LDL-c (mg/dl)	126 (109.50–147.50)	118 (101–141)	0.292
VLDL-c (mg/dl)	22.50 (17.25–32)	20 (15–28.50)	0.223
Non HDL (mg/dl)	154 (135–169)	136 (120–172)	0.108
AC	3.02 (2.65–3.46)	3.09 (2.78–3.77)	0.318
(TG/HDL) ratio	2.16 (1.64–3.23)	2.19 (1.59–4.17)	0.835
AIP	0.35 (0.25–0.52)	0.35 (0.21–0.62)	0.867
Castelli Index 1 (CRI-I)	4.02 (3.66–4.47)	4.09 (3.77–4.77)	0.318
Castelli Index 2 (CRI-II)	2.48 (2.14–2.82)	2.63 (2.26–3.09)	0.248

HDL- c: High density lipoprotein cholesterol, LDL- c: Low density lipoprotein cholesterol VLDL-c: Very low density lipoprotein cholesterol, AIP: Atherogenic Index of Plasma

AC: Atherogenic Coefficient

EPDS: Edinburgh Postnatal Depression Scale, PPD: Postpartum depression

p<0.05 is significant

*statistical significance using Mann Whitney U test

### Correlation between anthropometric measures, serum lipid parameters and EPDS score

Correlation of age, BMI and the lipid parameters were assessed using Spearman correlation coefficient and are represented in Table 4. The result showed a significant positive correlation between BMI and EPDS in non-PPD women (p = 0.004) and significant negative correlation between BMI and EPDS in women with PPD (p = 0.047). The current study also found a significant negative correlation between HDL-c and EPDS scores in postpartum depressed group (r = -0.140, p = 0.05), linking its relationship with development of postpartum depressive symptoms. Other serum lipid parameters were not observed to be correlated with EPDS scores in both postpartum depressive cases and control groups.

**Table 4:** Correlation analysis of parameters with EPDS score in non-PPD and PPD women.

Parameters	EPDS score			
	Non-PPD women (N = 250)		PPD women (N = 186)	
	Correlation coefficient (r)	P value	Correlation coefficient (r)	P value
Age (yrs)	0.094	0.137	0.068	0.355
BMI (kg/m^2^)	0.187	0.004*	-0.146	0.047*
T.Cholesterol (mg/dl)	0.097	0.127	-0.085	0.249
TGL(mg/dl)	0.070	0.274	-0.057	0.439
HDL-c (mg/dl)	0.050	0.434	-0.140	0.054*
LDL-c (mg/dl)	0.082	0.195	-0.005	0.943
VLDL-c (mg/dl)	0.066	0.300	-0.092	0.211
Non HDL(mg/dl)	0.097	0.125	-0.044	0.546
AC	0.039	0.540	0.080	0.276
TG/HDL ratio	0.032	0.610	-0.008	0.912
AIP	0.003	0.969	-0.011	0.882
Castelli Index 1 (CRI-I)	0.058	0.363	0.082	0.268
Castelli Index 2 (CRI-II)	0.022	0.733	0.108	0.143

HDL- c: High density lipoprotein cholesterol, LDL- c: Low density lipoprotein cholesterol VLDL-c: Very low density lipoprotein cholesterol, AIP: Atherogenic Index of Plasma

AC: Atherogenic Coefficient

EPDS: Edinburgh Postnatal Depression Scale, PPD: Postpartum depression

* Statistical significance using Spearman correlation analysis

## Discussion

Postpartum depression is a clinical condition defined as a depressive episode beginning within four to six weeks post-delivery, nevertheless the symptoms may last for up to a year [30]. A depressed mood in addition to sleep and appetite disturbances, loss in coherence to normal activities, loss of energy, and feelings of guilt and suicide thoughts may be present which is particularly challenging in differential diagnosis [31]. Since PPD is both under diagnosed and ineffectively treated, efforts for detection of this condition are of public health importance [32].

In the present study, the overall proportion of PPD was found to be 27% at six week postpartum. Findings from other community and hospital based studies in South India, reflect variability in the estimates of PPD with the reported prevalence of 18.6% and 13.8% [4, 33]. In our present study, we found that more women in the depressed group were employed and most of the women with depression reported marital disharmony and lack of support from the husband as shown in S1 Table. Factors like trouble sleeping, infant gender dissatisfaction, expectation of male baby, unwanted and pre-term pregnancies have contributed to maternal depressive symptoms in our current study. Our study also found that postpartum depression was related to prenatal anxiety, fear about labour during the prenatal period, mode of nursing the child such as use of infant formula as well as combination of both infant formula and breast feeding.The PPD rate was also observed to be significantly higher in women who had infants with illness compared to the non PPD women. However, our present study could not find significant association between family structure, socioeconomic status and obstetric factors like mode of delivery with depression in postpartum women.

In our present study, we estimated the serum levels of the lipid in postpartum women with and without depressive symptoms at six week post-delivery. In this cross sectional study of 186 postpartum women with depressive symptoms, no significant difference in serum lipid levels was observed. To the best of our knowledge, only one study on postpartum lipid levels is available and there are no studies on the correlation of serum lipid levels in mood disorders such as postpartum depression. Previous research reports have shown potential variation in the levels of serum lipids during pregnancy and after delivery [34]. Studies have shown increase in serum lipid levels such as TG, HDL, LDL and TC during the second and third trimester of pregnancy [35, 36]. Previous studies have shown that maternal serum lipids were elevated during pregnancy and remain above pregnancy levels after delivery except for triglycerides which decreases after delivery to pre-pregnancy levels [6]. Meanwhile, multiple studies have noted the effect of elevated lipid levels especially cholesterol and LDL-c and decreased level of triglyceride during puerperium [37, 38]. Several studies have assessed the relationship of lipid levels with depressive and anxiety disorders with mixed results. Previous studies have examined a positive association between HDL-c, LDL-c, VLDL-c and TC with depression. A study conducted among 604 Australian adult populations showed an association between increased serum cholesterol and symptoms of depressive mood [39]. In another study, a sample size of 300 healthy middle aged Swedish women, reported an association between low cholesterol levels and higher prevalence of depressive symptoms [40]. Other results reported negative association between depression and different lipid fractions. In a prospective study conducted among 168 participants of both gender Southern Taiwan population, an inverse relationship between serum lipids and major depression with different clinical subtypes. In contrast, another study by Rice MC et al among 131 community dwelling adults found that serum levels of LDL-C was inversely related to depressive symptoms among women but not men [41, 42]. A previous study reported by Lehto SM etal among 176 Finnish general populations observed significantly lower serum cholesterol (TC) and HDL cholesterol levels than normal control subjects, but no relation found between triglycerides with depression [43]. Similar results of significance with serum cholesterol and serum triglycerides with panic disorder and major depression disorder as reported by Hamidreza et al in a descriptive analytic study conducted 100 major depressive patients in Irani population [44].

The present result revealed no significant difference between serum levels of lipid parameters in PPD and non PPD women (Table 1).Western countries use the EPDS cut- off of 12 or above 12 for the diagnosis of PPD compared to the EPDS cut- off of 10 in the Indian scenario. In our current study, we used the EPDS cut- off of 10 for assessing depressive symptoms in postpartum women which could be the reason for no observable significant difference between serum lipid levels in PPD and non PPD women. These results were in concordance with the research findings of one of the earliest studies on Western populations of 43 postpartum women by Potter and Nestle [45]. Additionally, previous studies also explained that triglycerides decreases rapidly to pre-pregnancy levels after delivery. Lipid profile alters during pregnancy in women. Van Stiphout et al reported elevated levels of maternal serum lipids TC, HDL-c above pregnancy levels after delivery except for triglycerides [6].

In our current study, we calculated the different combinations of lipid parameters (atherogenic indices) such as Atherogenic Index of Plasma (AIP) and Castelli Risk Indexes (CRI), atherogenic coefficient (AC), TG/HDL-c ratio as markers of lipid atherogenic risk which were very useful in cardiovascular risk prediction. It has been observed that there were no significant differences in the atherogenic indices between the two groups. In our current study, we evaluated the role of these indices for assessing atherogenic risk in PPD women. Additionally, no previous studies have focused on the use of atherogenic indices for relating to mood disorders, instead examined the lipoprotein components or fractions so far [46, 47].

Another principal finding of this study is, subgroup analysis of serum levels of lipid parameters in PPD women based on their EPDS scores demonstrated that women with EPDS score of more than 12 were found to have significantly lower HDL-c levels than less EPDS score group. Additionally, Castelli risk indexes 1 & II levels were found significantly higher in PPD women with EPDS score >12 than those PPD women with EPDS score ≤ 12 (Table 2) when they were categorized based on EPDS score cut-off of 12. This is in concordance to the study results of Vargas et al reported higher levels of Castelli risk indexes 1 & II in patients with major depression [48]. Similarly, few researches underlined the atherogenic indices as risk for CVD in various psychiatric diseases which is the most common cause of female morbidity and mortality [49]. On the other hand, both lower and higher values of these ratios were found in patients with major depressive disorders [50]. We observed increased levels of both ratios in PPD women with EPDS score more than 12 irrespective of age and low BMI, when compared to PPD women with EPDS score less than 12. This observation of higher ratios in PPD women with increasing EPDS score is because of the decrease in HDL-c levels and not the increase in TC or LDL-c levels. However, the exact mechanism of the involvement of lipids in the pathogenesis of postpartum depression is not known yet. Previous studies have suggested the possible role of lipids in pathogenesis of depression and mood disorders. Lipids were found to be involved in the neuronal function of the brain, influencing perception and emotional behaviours [51]. Evidences support that the membrane forming lipids in the brain also contributes to pathogenesis of depression and mental disorders through the localization of membrane proteins thereby regulating its effect on neuronal function and neurotransmitter signalling [52]. Other possible mechanisms of lipids on depressive or anxiety related behaviours were the dysfunction of stress mediating neuropeptide [53] or neurotropic factors [54] as well as by the deregulation of the hippocampal neurogenesis [55].

These results persuade us to examine whether there is any variation in the levels of lipids among PPD women with and without suicidal ideation. Previous review reports noted the prevalence of postnatal suicidal thoughts in different settings, 4% in Finland to 15% in India [56]. It has been previously reported, suicide as one of the leading cause of maternal death at the post natal period within the six months of delivery [57]. On the other hand, maternal suicidal ideation has found to have negative impact on mother-infant relationship and reduced responsiveness to infants [58].We observed significantly lower levels of TC and HDL in women with suicidal ideation compared to women without suicidal ideation (Table 3).This is in concordance with the research reports of Zhang etal, on general population of US using a mental disorder diagnostic interview found low serum HDL-c levels were significantly associated with high prevalence of suicide attempts in women [59]. These changes of serum cholesterol levels in women with suicidal ideation occurred due to HDL-c levels rather than TC or LDL-c [60]. Several other case control studies corroborated our findings, an inverse relationship between TC and mood disorders particularly in major depressive disorders among European general populations [61]. These findings support the earlier studies conducted among US adults that higher levels of atherogenic indices are associated with higher scores of depressive symptoms measured by Centre for Epidemiologic Studies Depression (CES-D) scale [62]. The exact mechanism of correlation between serum cholesterol levels and suicidal ideation is not still completely elucidated. The plausible mechanism has been proposed which includes the reduction of serotonin activity [63], activity of the fatty acid particularly docosahexaenoic acid [64] and the anti-inflammatory activity of HDL-c [65]. However, our present study did not estimate the inflammatory activity of HDL-c to analyse the direct relationship between serum HDL-c levels suicidal ideation in depressed postpartum women.

Interestingly, we observed a significant positive correlation between BMI and EPDS scores in control group (r = 0.187, p = 0.004) and a negative correlation between them in postpartum depressed group (r = 0.146, p = 0.047). Several research studies have provided the evidence of association between BMI and PPD. In a prospective study by Carter et al, observed positive association between BMI and depressive symptomology at 4 and 14 months after delivery [66]. Similarly, postpartum weight retention was found to be associated with risk for obesity one year postpartum [67] and Sharon etal observed a strong association between the onset of postpartum depression and weight retention in the first year of postpartum [68]. In contrast, Linde et al observed reduced BMI to be associated with postpartum depressive symptoms and lost less than half as much weight as women without depression [69]. Similar to these findings, previous work by Karen Ertel et al evaluated the relationship between pre-pregnancy BMI and depressive symptoms, with lower odds among women with higher depressive symptoms [70]. Also, no significant association between BMI and depressive symptoms in women after delivery was also observed [71, 72]. Complementing this finding, the higher levels of lipid in non-PPD and lower levels in PPD group substantiate the reason for altered BMI. However, the exact endocrinologic factors involved in this variation need further research.

A recent study Farias et al, exploring the effect of pre-pregnancy BMI on lipid metabolism throughout pregnancy, observed higher serum levels of lipids in pre-pregnancy women with overweight and obese BMI compared to normal weight pre-pregnancy woman. Also, pre-pregnancy obese women has been shown to have lower rate of change of serum lipid levels such as TC, LDL-c, TG and slight increase in serum HDL-c levels compared to normal weight pregnant women [73]. Pre-pregnancy overweight or obesity status has been found to be associated with lower depressive symptoms during pregnancy period [70].We observed similar results of negative association of BMI with depressive symptoms in PPD women although further follow-up studies are needed to substantiate the findings of these reports. These findings point towards the BMI dependent association of serum lipid levels in postpartum depression.

Our study observed a significant negative correlation between HDL-c and EPDS scores in PPD group, implicating low serum levels of HDL-c as a marker for the development of postpartum depressive symptoms in PPD women. Though the study did not observe any significant variations in other lipid levels between the study groups irrespective of lower BMI in the PPD group, low HDL-c and TC levels seem to be important factors in defining their association in the development of PPD. However, maternal age and lipid indices did not contribute to any statistical significance, probably due to relatively narrow age groups as well as the ethnicity of our population. Notably, earlier studies have implicated no association of postpartum lipids with maternal age or weight which matches our findings [74]. On the other hand, previous evidences reported association between BMI and depression with conflicting results. Contradictorily, other studies demonstrated both older age and higher BMI are associated with elevated lipid levels [75].

## Conclusion

In conclusion, our study demonstrates that low levels of serum HDL-c is associated with higher depressive symptoms in women at six week post- delivery, implicating its association in the pathophysiology of PPD. Castelli’s Risk Indices are observed to be increased in PPD women with higher EPDS score because of the decrease in HDL-c and not due to increase in TC. Additional studies based on large sample size with methodology variation, postpartum period comparison may reveal the role of serum cholesterol in severe postpartum depressive symptomology may generate biological targets for future novel therapeutic strategies.

## Limitations

EPDS that has been used in our study to screen for postpartum depression at six week post-delivery has moderate sensitivity and specificity. Although this scale is validated, there is possibility of misclassification of depression as this instrument only screen for the presence or absence of depressive symptoms and we accept this as the limitation of our study.

## Supporting information

S1 Table Comparison of socio demographic, Obstetric and infant characteristics of Non- PPD and PPD women. (DOCX)
