# Supplementary material for: Low serum levels of High-Density Lipoprotein cholesterol (HDL-c) as an indicator for the development of severe postpartum depressive symptoms
Source: PLoS One. 2018 Feb 14;13(2):e0192811. doi: 10.1371/journal.pone.0192811 (PMC5812627; doi:10.1371/journal.pone.0192811)
Supplement: S1 Table — (DOCX) [file pone.0192811.s001.docx]

**Supplementary Data table**

**S1 Table : Comparison of socio demographic, Obstetric and infant characteristics of Non- PPD and PPD women**

| **Variables** | | **Characteristics** | **Total(N=436)** | | | **Non- PPD women**  **N=250 (%)** | **PPD women**  **N=186 (%)** | | **P Value** | | |
| --- | --- | --- | --- | --- | --- | --- | --- | --- | --- | --- | --- |
| Education | | No education  Elementary  Higher secondary  College  Professional degree | 14(3.2)  88(20.2)  154(35.4)  87(20)  92(21.1) | | | 8(3.2)  50(20)  96(38.4)  47(18.8)  49(19.6) | 6(3.2)  38(20.5)  58(31.4)  40(21.6)  44(23.2) | | | 0.627 | |
| Current Employment Status | | Employed  Unemployed | 102(23.4)  334(76.6) | | | 46(18.4)  204(81.6) | 56(30.3)  130(69.7) | | | **0.003*** | |
| Family Structure | | Nuclear  Extended | 133(30.5)  303(69.5) | | | 74 (29.6)  176(70.4) | 59(31.7)  127(68.3) | | | 0.674 | |
| Socioeconomic status | | Low  Moderate  High | 251(57.7)  98(22.5)  86(19.8) | | | 144(57.6)  63(25.2)  43(17.2) | 107(57.8)  36(19)  43(23.2) | | | 0.148 | |
| Marital disharmony | | Yes  No | 35(8)  401(92) | | | 6(2.4)  244(97.6) | 29(15.6)  157(84.4) | | | **0.000*** | |
| Stressors or life events | | Problems with MIL, Husband  Financial issues /others  Problems with parents  No events | 102(23.4)  35(8)  14(3.2)  285(65.4) | | | 39(15.6)  13(5.2)  7(2.8)  191(76.4) | 63(33.9)  22(11.8)  7(3.8)  94(50.5) | | | **0.000*** | |
| Infant gender  dissatisfaction | | Yes  No | 76(17.4)  360(82.6) | | | 32(12.8)  218(87.2) | 44(23.7)  142(76.3) | | | **0.002*** | |
| Sleep disturbances after delivery | | No  Bad dreams regularly  Pain  Breathing discomfort /Feeling too hot/cold  Erratic sleep pattern of baby | 201(46.1)  29(6.7)  89(20.4)  92(21.1)  25(5.7) | | | 133(53.2)  14(5.6)  54(21.6)  27(10.8)  22(8.8) | 68(36.6)  15(8.1)  35(18.8)  65(34.9)  3(1.6) | | | **0.000*** | |
| Current pregnancy | | Planned  Unplanned  Unwanted | 305(70)  126(28.9)  5(1.1) | | | 187(74.8)  61(24.4)  2(0.8) | | 118(63.4)  65(34.9)  3(1.6) | **0.035*** | | |
| Mode of delivery | | Vaginal  Caesarean  Forceps  Vacuum assisted | 317(72.7)  102(23.4)  12(2.8)  5(1.1) | | | 184(73.6)  55(22)  10(4)  1(0.4) | | 133(71.5)  47(25.3)  2(1.1)  4(2.2) | 0.082 | | |
| Type of delivery | | Term  Pre-term | 395(90.6)  41(9.4) | | | 227(90.8)  23(9.2) | | 168(90.3)  18(9.7) | 0.866 | | |
| Prenatal anxiety/fear about labor | | Yes  No | 225(51.6)  211(48.4) | | | 92(36.8)  158(63.2) | | 133(71.5)  53(28.5) | **0.000*** | | |
| Mode of nursing the child | | Breast milk  Infant formula  Both | 426 (97.7)  6(1.4)  4(0.9) | | | 250(100)  0(0)  0(0) | | 176(94.6)  6(3.2)  4(2.2) | **0.001*** | | |
| Infant illness | Yes  No | | | 109(25)  327(75) | 50(20)  200(80) | | | 59(31.7)  127(68.3) | **0.005*** | |  |
| Infant temperament | Easy going  Slow to warm up  Difficult ,no EC  Difficult ,EC | | | 306(70.2)  51(11.7)  57(13.1)  22(5) | 195(78)  19(7.6)  32(12.8)  4(1.6) | | | 111(59.7)  32(17.2)  25(13.4)  18(9.7) | **0.000*** | |  |
